# Recent advances in radiotherapy

**DOI:** 10.1186/1741-7015-8-25

**Published:** 2010-04-28

**Authors:** SA Bhide, CM Nutting

**Affiliations:** 1 Institute of Cancer Research and Royal Marsden Hospital, Fulham Road, London, UK

## Abstract

Radiation therapy has come a long way from treatment planning based on orthogonal radiographs with large margins around tumours. Advances in imaging and radiation planning software have led to three-dimensional conformal radiotherapy and, further, to intensity modulated radiotherapy (IMRT). IMRT permits sparing of normal tissues and hence dose-escalation to tumours. IMRT is the current standard in treatment of head and prostate cancer and is being investigated in other tumour sites. Exquisitely sculpted dose distributions (increased geographical miss) with IMRT, plus tumour motion and anatomical changes during radiotherapy make image guided radiotherapy an essential part of modern radiation delivery. Various hardware and software tools are under investigation for optimal IGRT.

## Introduction

The delivery of radiotherapy has changed significantly over the last few decades. We have moved from conventional radiotherapy using simple rectangular treatment fields to increasingly conformal radiotherapy techniques such as three dimensional conformal radiotherapy (3D-CRT) and intensity modulated radiotherapy (IMRT). These changes in the delivery of radiotherapy have come about as result of trying to improve the delivered dose to tumour bearing tissues, and reduce irradiation of organs at risk (OARs), hence improving the therapeutic ratio of the radiation treatment. The radiation dose is prescribed to the planning target volume (PTV), which includes the gross tumour volume (GTV) plus areas of microscopic spread (clinical target volume, CTV) and a margin around it to account for the systematic and random errors plus physiological organ changes that occur during the treatment planning and delivery process [1,2]. Using three-dimensional conformal radiotherapy to deliver a radical dose to the PTV, results in a significant dose to the OARs. There is robust evidence from several tumour sites, such as head and neck, prostate and lung, supporting dose-escalation and/or altered fractionation for improved outcomes [3-6]. Reducing the dose to the OARs using techniques such as IMRT and reducing the size of the PTV using image guided radiotherapy (IGRT) enables radiation dose-escalation to be done, to improve the treatment outcomes.

## IMRT

IMRT is an advanced approach to three-dimensional treatment planning and conformal therapy. It optimizes the delivery of irradiation to irregularly-shaped volumes and has the ability to produce concavities in radiation treatment volumes. IMRT can be delivered using linear accelerators with static multi-leaf collimators (MLC, step and shoot IMRT) or dynamic leaf MLCs, tomotherapy machines or volumetric arc modulated therapy (VMAT). The simultaneous boost IMRT allows for varying doses to be delivered to various target volumes in a single phase and obviates the need for field matching and the use of electrons, thus minimizing the dosimetric uncertainties.

When treating head and neck cancers, IMRT allows for a greater sparing of normal structures such as salivary glands, upper aero-digestive tract mucosa, optic nerves, cochlea, pharyngeal constrictors, brain stem and spinal cord [7-9]. Salivary gland sparing using IMRT in various head and neck sub-sites has been demonstrated in randomized and non-randomised trials. The multi-centre study (PARSPORT), that compared parotid sparing IMRT with standard radiotherapy in patients with oropharyngeal and hypopharyngeal cancer, showed a significant reduction (40% versus 74%) in the rate of grade 2 xerostomia (LENT-SOMA) in the IMRT arm at 1 year post-radiotherapy [10]. IMRT also enables the sparing of the pharyngeal constrictor muscles, which are important for a normal swallow, and therefore has the potential to reduce acute and late radiation induced dysphagia [9]. By virtue of its ability to spare the cochlea, IMRT has the potential to reduce the incidence of radiation induced hearing loss [11]. The significant increase in the burden of toxicity resulting from radiotherapy can be reduced using IMRT. The reduction in the normal tissue toxicity permits dose-escalation to improve outcomes.

Escalation of radiation dose to greater than 68 Gy using hypofractionation has been shown to improve outcomes in localized carcinoma of the prostate [4,12]. The dose to the prostate is limited by gastro-intestinal and genitor-urinary tolerance. The use of IMRT has resulted in the safe delivery of hypofractionated escalated doses to the prostate with reduced acute and late rectal and bladder toxicity [6,13,14]. In patients with a high risk of pelvic lymph node metastases, pelvic nodal irradiation improves outcome [15]. The prostate, seminal vesicles and the pelvic nodes can be treated using IMRT, with acceptable gastro-intestinal and genitor-urinary toxicity [16,17].

In gynaecological cancers, whole pelvic radiotherapy provides improved outcomes but at a cost of increased gastro-intestinal and haematologic toxicity. IMRT has been shown to reduce the acute and late toxicity without affecting treatment outcomes [18-21].

The dosimetric benefit of IMRT in sparing OARs has been proven in cancer of pancreas and stomach (liver, kidneys, spinal cord and small bowel) and anus and rectum (small bowel, bladder and pelvic bone marrow). However, there is limited clinical data on the outcomes in terms of tumour response and late normal tissue toxicity. Trials are currently underway to address this issue [22].

IMRT can be used when delivering post-operative radiotherapy for breast cancer as single phase or for boost after 3D-CRT. This is of particular value in women with large or irregular breasts and improves late cosmesis and reduces the dose to the heart and the lung [23-25].

## IGRT

The sharp dose gradients that exist with IMRT plans could result in a geographical miss of the tumours or overdose to the OARs. Optimal IMRT delivery therefore relies on accurate image guidance. In tumours which exhibit a large physiological motion the margins around the CTV can be quite large. Reduction in this margin allows for a reduction in dose to the organs at risk. This gives a scope for an improvement in the therapeutic ratio by altering the dose, dose per fraction and also dose-escalation to take advantage of the steep portion of the radiation dose response curve. IGRT is a useful tool that can detect and correct random and systematic errors that occur during treatment delivery. Portal imaging and digitally reconstructed radiographs is a basic form of IGRT. More advanced IGRT techniques are being introduced in clinical practice which allow for target oriented positioning as opposed to the patient oriented positioning that is currently used. Image guidance can be used for improved tumour delineation and/or to correct for intra and/or inter-fraction motion during radiotherapy.

### Image guidance for delineation

Computed tomography (CT) scans are the standard imaging modality used in radiation treatment planning as they provide a three-dimensional view of the tumours and normal anatomy, along with the electron density data which enables dose calculations. However CT scans are inferior to magnetic resonance imaging (MRI) scans in the detailed definition of soft tissues (microscopic tumour extension) and tissue planes and can be affected by artifacts such as dental amalgam and hip arthroses. CT-MRI fusion should be considered for radiotherapy planning wherever possible, especially in central nervous system and skull base tumours. Positron emission tomography (PET) enables biological imaging of tumours. Initial studies using [(18)-F] fluoro-2-deoxy-D-glucose positron emission tomography (FDG-PET), which highlights the proliferating areas of the tumour, have been reported [26]. These have shown that FDG-PET can aid delineation of the GTV and PET guided dose escalation using IMRT [27-32]. Hypoxic regions of tumours are radio resistant and increasing the radiation dose might help overcome the radioresistance. PET scanning using two radioactive tracers, namely fluorine-18-labelled fluoromisonidazole (F-MISO) and copper(II)-diacetyl-bis(N(4)-methylthiosemicarbazone) (Cu-ATSM) have been shown to highlight the hypoxic areas of tumours. Preliminary studies escalating the radiation dose to the hypoxic areas have demonstrated the feasibility of this approach in terms of acute toxicity [33,34]. The PET images could be fused with the planning CT scans and these could be used for biological dose optimization (as opposed to the currently used dose-volume hostogram based optimization) during inverse planning IMRT. However, follow-up data for outcomes and toxicity from larger studies using PET guided dose escalation are required before this approach can be used in standard clinical practice.

## IGRT for PTV margin reduction

Reduction in the size of the tumour and change in the local anatomy lead to an inter-fraction change in both the target volume to be treated and the OARs. Significant intra-fraction motion can occur when treating prostate, rectal, gynaecological cancers and head and neck tumours. Regular in-room images can be obtained using 'CT on rails', kilovoltage cone beam CT (kVCBCT), megavoltage cone beam CT (MVCBCT) or using a tomotherapy machine. CT on rails consists of a CT scanner in the treatment room, at the opposite end of the linear accelerator. kVCBCT consists of a kilovoltage X-ray tube combined with a flat panel image detector mounted orthogonally to the therapy X-ray beam on a linear accelerator. MVCBCT uses an electronic portal imaging device (EPID) mounted on the gantry and the mega-voltage photon beam for image generation. Tomotherapy uses a machine which integrates helical megavoltage CT with a linear accelerator. The images obtained from these in-room modalities are fused with the CT used for planning, using bony and soft tissue contrast and changes in the treatment plan or patient position, can be made to account for the inter-fraction motion. In addition, tumours can be tracked using infra-red markers placed on the patient's skin and aligning these to bony landmarks or fiducial markers in order to make changes to the treatment plan based on the changes in the internal anatomy [35,36].

Larger PTV margins have to be used for tumours of the lung and intra-abdominal tumours to allow for the motion during respiration. The development of four-dimensional CT with multi-slice detectors and faster image reconstruction has enabled image acquisition while the patient breathes. CT slices are obtained during every phase of the respiratory cycle, which are then combined to quantify the respiratory motion [37]. The four-dimensional CT can be used to generate PTV margins using the free-breathing technique, where the composite tumour volume over the entire breathing cycle is created and a margin is applied to allow for microscopic spread and set-up uncertainties. Alternatively, it can be used to deliver radiation using the breath-hold technique where the beam is switched on with the patients holding their breadth in the expiratory or the inspiratory phase [37]. In patients who find voluntary breath control difficult, a ventilatory gating approach can be used where the patient's chest movements are tracked using a fiducial marker on the body surface and the beam is switched on in the pre-determined phase of the respiratory cycle.

## Image guidance for treatment verification

Verification is a vital cog in the radiation treatment delivery cycle. Verification is undertaken before treatment starts, and regularly during treatment, and ensures that under-dosing to the tumour and over-dosing to the OARs is avoided by minimizing the systematic and random errors. Advanced forms of EPID have been developed. In addition to the conventional two-dimensional verification, modern devices also enable three-dimensional volumetric verification (using kVCBCT) and *in vivo *dosimetry. The CT- detector array systems on tomotherapy machines can also be used for verification and *in vivo *dosimetry [35]. Combining the information obtained from *in vivo *dosimetry of normal tissues and the monitoring of normal tissue toxicity (common toxicity criteria for adverse events, CTCAE) enables the calculation of normal tissue complication probabilities which would provide information on the therapeutic ratio of a particular treatment technique and aid setting dose constraints for IMRT.

## Stereotactic radiotherapy

Hypofractionated accelerated radiotherapy is thought to improve outcomes by reducing the impact of tumour cell repopulation. Stereotactic radiotherapy enables the delivery of exquisitely conformed radiation in large fraction sizes, which also enables improved tumour control while limiting the normal tissue toxicity. Due to the exquisite dose sculpting, robust image-guided technologies (gating or chasing) have to be coupled with the radiation delivery systems [38]. Stereotactic radiotherapy can be delivered using linear accelerator systems or Cyberknife^® ^(Accuray Inc, CA, USA). This technique is currently widely used for treating intra-cranial oligometastases [39]. There is increasing evidence supporting its use for the radical treatment of stage I lung cancer, renal cell carcinoma, hepato-cellular carcinoma, spinal tumours and prostate carcinoma (as a primary treatment or a post-treatment boost). It can also be used for treatment of lung and hepatic metastases [38].

## Particle therapy

Charged particles, such as protons, deposit little energy until they reach the end of their range (depending on their energy), at which point most of the energy is deposited in a small area known as the Bragg peak. This has advantages in terms of normal tissue sparing, better dose homogeneity and a reduced dose bath effect (low radiation dose to normal tissue). Intensity modulated proton therapy (IMPT) allows for the modulation of the fluence and the position of the Bragg peak, permitting three-dimensional dose distributions. There are no randomized trials comparing IMPT with IMRT. The delivery of proton therapy on a wide scale is restricted by the limited availability of proton therapy machines due to the high financial resources required. Newer technologies, such as laser-accelerated proton therapy, could replace the current cyclotron facilities for proton therapy, which will be a compact, cost-effective way to deliver energy-and intensity-modulated proton therapy (EIMPT) [40]. The current role of proton therapy lies in tumours close to the skull base, spinal cord and in paediatric patients [41] where proton therapy provides the maximum benefit in terms of normal tissue sparing.

## Future directions

The role of IMRT in normal tissue sparing is well established. Future research into the optimization of IMRT should focus on improvements in the time required for planning and delivery using techniques such as VMAT. Improvements in therapeutic ratio could be achieved by: reducing the low dose to normal structures (dose-bath effect) using techniques such as IMPT; investigating dose-escalation to tumours using biological volumes; and increasing the effective dose to tumours using altered fractionation (linac based or using stereotactic radiosurgery). Using imaging modalities, such as MRI and CT, for better treatment verification and development of sophisticated software solutions for online and off-line plan corrections would help to reduce errors during treatment delivery.

## Summary and conclusions

The last two decades have seen significant technological advances for radiation delivery in terms of more precise dose delivery using IMRT and IGRT. The result has been improved sparing of normal tissue and, hence, the potential to improve cancer outcomes using dose-escalation and/or altered fractionation. Last, but not least, none of the advances in radiotherapy planning and delivery would have been possible without the availability of faster, more powerful computer systems that enable the efficient running of the advanced softwares required for planning and delivery.

## Abbreviations

3D-CRT: three-dimensional conformal radiotherapy; CT: computed tomography; CTV: clinical target volume; EPID: electronic portal imaging device; FDG: fluorodeoxyglucose; GTV: gross tumour volume; IGRT: image guided radiotherapy; IMRT: intensity modulated radiotherapy; kVCB: kilovoltage cone beam; MLC: multi-leaf collimators; MRI: magnetic resonance imaging; MVCB: megavoltage cone beam; OAR: organ at risk; PET: positron emission tomography; PTV: planning target volume; VMAT: volumetric arc modulated therapy.

## Competing interests

The authors declare that they have no competing interests.

## Authors' contributions

SB did the literature search and wrote the manuscript. CMN made a critical appraisal of the manuscript for content and accuracy. Both authors read and approved the final manuscript.

## Pre-publication history

The pre-publication history for this paper can be accessed here:

http://www.biomedcentral.com/1741-7015/8/25/prepub
